# Understanding the Rapid Reduction of Undernutrition in Nepal, 2001–2011

**DOI:** 10.1371/journal.pone.0145738

**Published:** 2015-12-23

**Authors:** Derek D. Headey, John Hoddinott

**Affiliations:** 1 Poverty, Health and Nutrition Division, International Food Policy Research Institute, Washington DC, United States of America; 2 Division of Nutrition Sciences and Charles H. Dyson School of Applied Economics and Management, Cornell University, Ithaca, NY, United States of America; Hôpital Robert Debré, FRANCE

## Abstract

South Asia has long been synonymous with unusually high rates of undernutrition. In the past decade, however, Nepal has arguably achieved the fastest recorded decline in child stunting in the world and has done so in the midst of civil war and post-conflict political instability. Given recent interest in reducing undernutrition–particularly the role of nutrition-sensitive policies–this paper aims to quantitatively understand this surprising success story by analyzing the 2001, 2006, and 2011 rounds of Nepal’s Demographic Health Surveys. To do so, we construct models of the intermediate determinants of child and maternal nutritional change and then decompose predicted changes in nutrition outcomes over time. We identify four broad drivers of change: asset accumulation, health and nutrition interventions, maternal educational gains, and improvements in sanitation. Many of these changes were clearly influenced by policy decisions, including increased public investments in health and education and community-led health and sanitation campaigns. Other factors, such as rapid growth in migration-based remittances, are more a reflection of household responses to changing political and economic circumstances.

## Introduction

Scientists of many disciplines have long been puzzled as to why South Asian countries have such unusually high rates of malnutrition, especially relative to Africa south of the Sahara [1–7]. Since the term was first popularized in the mid-1990s, however, the nature of this *Asian enigma* has changed significantly. In the 1990s Nepal had the highest recorded rate of child stunting in the world, with around 60 percent of children younger than 5 years being stunted, many of them severely so. From 2001 to 2011 Nepal achieved the fastest recorded reduction in child stunting in the world, reducing child stunting from 56.6 to 40.0, a reduction of 1.66 points per year. Most remarkably, Nepal achieved this success without the stellar economic growth rates of China, India or Vietnam, and did so in the midst of a violent Maoist insurgency (2001–2006) and subsequent political instability and uncertainty (2006–2011).

In this paper we assess Nepal’s dramatic success through a quantitative analysis of three rounds of Demographic Health Survey (DHS) data (2001, 2006, and 2011). The paper is related to, but nevertheless distinct from, several strands of the extant nutrition literature. First, the aforementioned literature on the South Asian enigma has also attempted to identify the determinants of undernutrition in South Asia, but largely through static approaches such as Asia-Africa comparisons [3,7]. In contrast, this paper is a rare attempt to understand nutritional change over time. Second, there is indeed a small but somewhat diverse literature on nutrition success stories, which has largely been qualitative and focused on questions of policy and political process, including the important question of multisectoral nutrition efforts [8–11]. While such studies are important for understanding the deeper social and political drivers of nutritional change, qualitative and anecdotal case studies of success stories do not generate objective evidence on the contributions of different sectors. Third, there is a large body of literature focus on rigorously evaluating the nutritional impact of specific interventions, typically nutrition-specific interventions [12–13]. Though internally rigorous, control trials typically say little about the larger programmatic impacts over space or time. The literature, moreover, has much less emphasis on nutrition-sensitive interventions–in sectors such as education, sanitation, water and health–which can be very important for reducing undernutrition [13]. Hence our focus on understanding potential drivers of nutritional change at the national level complements experimental evaluation of nutrition-specific interventions.

The paper addresses two inter-related questions. The first is essentially static: Which factors best explain variation in nutrition outcomes among Nepalese children? The second is dynamic: which of these factors best predict change in nutrition outcomes over time? In terms of data, a key strength of our paper is that we are able to examine the predictive power of a wide range of policy-relevant explanatory variables (wealth, education, health service utilization, water supply, sanitation, demographic outcomes, and intergenerational transmission via maternal height), all of which are measured in nationally and subnationally representative surveys. With regard to methods we use nonparametric graphical techniques (which are particularly useful for exploring nonlinear relationships), pooled multivariate regression models, and simple decompositions in which nutritional change from a given variable is the product of the change in the mean of that variable and its regression coefficient at population means. A limitation of this approach is that the regression coefficients in our models cannot be interpreted as strictly causal effects: our interpretations instead focus on how well different indicators predictive nutritional change among Nepalese children and over time. Even so it is worth re-emphasizing that causal inferences rest on experimental designs that are simply not feasible to implement at the national level for different interventions in different sectors. Hence, understanding national level success stories requires some tradeoff between internal and external validity.

Nepal is a particularly interesting case study because it has managed to achieve extremely rapid nutritional improvements in spite of political and social turmoil. Despite reasonably strong growth household income (Table B in S1 Text), high levels of inequality across the country’s different regions and different social groups provided the catalyst for a Maoist insurgency (largely from 2000 to 2006) that resulted in 15,000 deaths and severe disruption to the economy. A peace agreement brokered in 2006 ended the conflict but resulted in a painfully slow transition to a new constitutional democracy. On the social front, however, change has been far more positive. The share of the government budget devoted to education rose steadily from around 10 percent in 1988–1992 to almost 20 percent in 2006–2011 [14]. Since the late 1990s, reforms in the health sector led to a sizeable increase in the budget to around 7 percent of total public spending and a realignment toward primary care at the community level, with much greater involvement of nongovernmental organizations and local governments [15–16]. By the late 2000s the government had established 15,000 primary healthcare outreach clinics staffed by volunteer health workers who provided grassroots health services, leading to significant improvements in immunizations, vitamin A supplementation, prenatal, neonatal and postnatal care (including nutritional advice), and treatment of common diseases, particularly diarrhea, malaria, and acute respiratory infections [17–20]. Nepal also saw vast improvements in sanitation (and to a lesser extent, water), with significant adoption of a Community-Led Total Sanitation (CLTS) approach built around community-based behavioral change and construction of low-cost toilet facilities [21]. From 2001 to 2011 the percentage of households engaged in open defecation fell from 75.1 percent to 42.3 percent.

Perhaps unsurprisingly, then, we find indicative evidence that nutritional change in Nepal has been driven by a very multifaceted process. Improvements in child growth scores and stunting rates are strongly associated with health and nutrition interventions, particularly utilization of antenatal and neonatal care, which have expanded rapidly over time. Maternal education gains are a second major factor predicting sizeable nutritional gains. Wealth accumulation is a third important factor, but one more difficult to link to specific policies or specific pathways. Rapid improvements in sanitation—particularly the dramatic reduction in open defecation—are a fourth factor, and one likely linked to community sanitation campaigns. Others—such as improved family planning outcomes and intergenerational transmission (maternal height)—generally played a much smaller role. Finally, our models generally perform very well at explaining nutritional change over time, predicting approximately 80 percent of the actual change in HAZ and stunting over 2001–2011.

## Material and Methods

To explain nutritional change over time we analyze three rounds of Nepal’s DHSs [18–20]: 2001, 2006, and 2011. These surveys are well suited for our purposes. They are multi-purpose survey instruments are nationally representative (and sub-nationally representative) and highly consistent over time in their coverage of a wide array of nutrition-relevant indicators.

As our dependent variable we focus on HAZs for preschool children (0–59 months) as measured using the World Health Organization growth standards described [22]. Linear growth is a very relevant indicator of overall nutrition, and the reduction in stunting (HAZs of two standard deviations or less) is the standard metric of long term nutritional success. However, epidemiologists argue against the analysis of dichotomous rather than continuous variables on the grounds that dichotomizing variables unnecessarily weakens the power of statistical tests [23, 24]. In our case our pooled sample size is large enough to greatly reduce this concern, so we also analyze rates of moderate stunting (HAZ < –2) and severe stunting (HAZ < –3).

Most of our explanatory variables are straightforward inclusions in nutrition models (Table 1). That said, we note the following.

**Table 1:** Variable definitions.

Short name	Definition
HAZ	Height-for-age *z* score (HAZ) measured against World Health Organization (2006) norms
Stunting	HAZ < –2
Severe stunting	HAZ < –3
Asset index (1–10)	6-component index; see text and Appendix A for details
Maternal education (years)	Mother’s years of education
Paternal education (years)	Father’s years of education
4 or more ANC visits	Dummy = 1 if mother received 4 or more antenatal care (ANC) visits
Iron during pregnancy	Dummy = 1 if mother received iron supplements during pregnancy
Born in hospital (0/1)	Dummy = 1 if child was born in hospital or medical clinic
All vaccinations (0/1)	Dummy = 1 if child received LPG; polio (2 shots); diphtheria, pertussis, and tetanus (3 shots); and measles vaccines
Preceding birth interval (years)	Interval between birth of present child and any previous child
Open defecation (%, village)	Percentage of households in a village not regularly using a latrine
Water—tubewell (0/1)	Dummy = 1 if household water was sourced from tubewell
Water source—piped (0/1)	Dummy = 1 if household water was sourced from pipes
Women's empowerment (0–1)	Equally weighted index of women’s involvement in 4 household decisions
Maternal height (cm)	Mother’s height (in centimeters)

Source: Authors’ construction.

First, we followed Filmer and Pritchett [25] in using a DHS asset index to proxy for household wealth. These and other authors have shown that these asset indexes are good proxies for household socioeconomic status in terms of sharing strong correlations with other welfare indicators, including child nutrition outcomes. For the three DHS rounds in our analysis we use a pooled principal components analysis to construct an index comprising six indicators (see Supporting Information). These indicators, and their respective time-invariant factor loadings, were radio ownership (0.15), TV ownership (0.50), bicycle ownership (0.22), use of improved cooking fuel (kerosene, biogas, electricity; 0.46), basic flooring (–0.49), and household access to electricity (0.47). After applying these loadings as weights in the index, we then rescaled the index to vary between a minimum score of 0 and a maximum score of 10.

This relatively parsimonious index performs well in explaining nutrition outcomes and is highly correlated with more sophisticated indexes based on a larger set of asset indicators that were only available in the 2006 and 2011 rounds (S1 Text). A limitation of this measure of household wealth is that it contains several variables that are influenced by activities outside the home (for example, public electricity supply) and contains variables that might have direct effects on nutrition in addition to contributing to household wealth. For example, TVs and radios carry programs with prominent health and family planning messages [20]. Hence the wealth effects in our models could include multiple pathways.

A second issue pertains to our own construction of the percentage of households in a village (DHS cluster) engaged in open defecation. While many previous studies measure this indicator at the household level, Spears [7] points out that open defecation is a negative externality, especially since household members are substantially immune to much of the bacteria in their own waste. This implies that community-level indicators of open defecation are more relevant than household-level variables.

Finally, we construct a decisionmaking index for women as an indicator of women’s empowerment. DHS asks a series of questions about whether a woman has a say in large household purchases, her own healthcare, the spending of money she has earned, and visits to relatives. We construct an index attaching equal weights to all four variables, with a score of 1 signifying involvement in all four decisions and 0 no involvement.

We use linear regression models and linear probability models (LPMs) in STATA^TM^ (S4 Text) to assess the associations between nutrition outcomes (*N*) for a child i at time t and a vector of time-varying intermediate determinants (***X***), a vector of largely time-invariant control variables (child and maternal age dummies, location fixed effects; ***μ***_***i***_), and trend effects represented by a vector of year dummy variables (***T***) to control for any unobserved time-varying national factors that might influence nutrition (including any different in surveys). The vector of coefficients (***β***) on **X** constitutes the set of parameters of principal interest. With the addition of a standard white noise term (*ε*_*i*,*t*_) we represent this relationship by Eq 1:

$$N_{i,t}=\beta X_{i,k}+\mu_{i}+T+\varepsilon_{i,t}$$ (1)

Apart from the standard least squares assumptions, there are several misspecification issues to consider in estimating Eq (1). First, we would ideally like to identify policy-driven (supply-side) determinants of nutrition outcomes, particularly in the domains of health, sanitation, and family planning outcomes. Appropriate control for household wealth and parental education would presumably take us a long way to purging the regression of demand-side factors that might simultaneously influence nutrition and the demand for health, sanitation, and family planning services, but such an assumption is impossible to test. Hence we re-emphasize that the evidence we generate on policy-relevant factors is circumstantial.

A second important assumption in Eq (1) is that the model is linear. To that end we took two steps. First, we adopted a flexible specification of the time-invariant determinants of nutrition including monthly dummy variables to capture the progressive growth-faltering process that malnourished populations undergo until around two years of age [26]. Second, we undertook nonparametric graphical analyses of all time-varying continuous variables to examine whether nonlinearities exist in their relationships with HAZs. There is minimal evidence of any significant nonlinearities, however (Fig C in S2 Text).

Eq (1) is used to answer the first of our two questions about the determinants of undernutrition in Nepal but also can be used to understand drivers of nutritional change over time. Under the assumption that the ***β*** coefficients are time-invariant and the error term has a mean of 0, the first difference of Eq 1 between time 1 and time K is given by the following:

$${\Delta \bar{N}}_{i,t}=\beta ({\bar{X}}_{t=K}-{\bar{X}}_{t=1})$$

where bars represent sample means.

Note that if the coefficients are time varying, an Oaxaca-Blinder decomposition [27] can be used to disaggregate the estimated change in the dependent variable into changes in endowment, changes in coefficients, and interactions between the two (if there is a high degree of parameter stability across time then the two decomposition techniques are equivalent). In contrast to using all three rounds of data, the Oaxaca-Blinder decomposition uses only the first and last rounds and tests for systematic coefficient differences between the two rounds. We opt for the linear decomposition described in the text because our preferred regression models tend to perform less well in the 2011 round when the survey measured nutrition outcomes for a much smaller sample size (every second household only). We suspect that the much smaller sample size in this round produces this instability, rather than any genuine change in the effects (coefficients) of the explanatory variables.

## Results and Discussion

**Fig 1:**
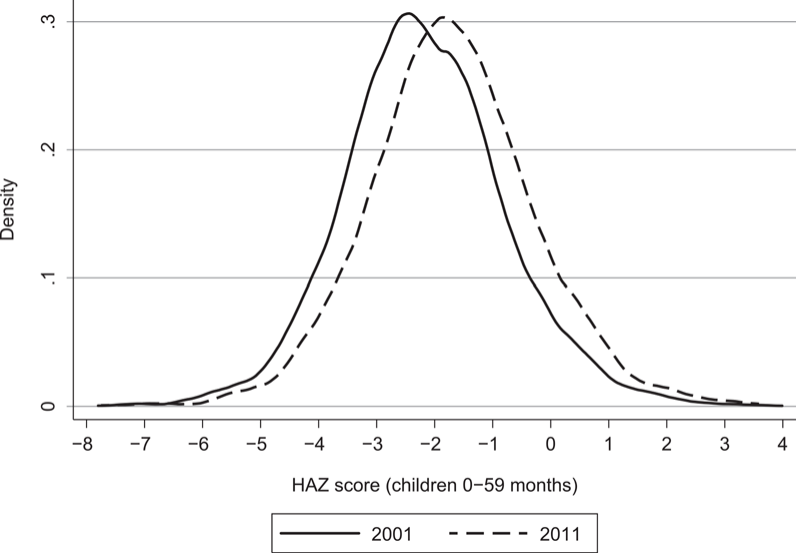
Shifts in the distribution of height-for-age *z* scores (HAZs), 2001 to 2011. Source: Kernel density estimates from the Demographic Health Surveys [18,20].

**Fig 2:**
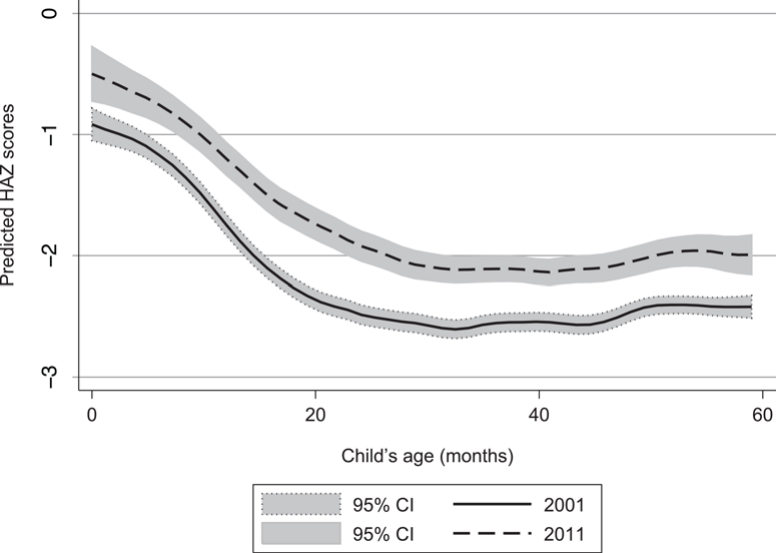
Shifts in height-for-age *z* scores (HAZs), by child’s age, from 2001 to 2011. Source: These are local polynomial smoothing predictions with 95% CIs estimated from the Demographic Health Surveys [18,20]. Note: CI = confidence interval.

Fig 1 shows the kernel density estimations of the distribution of child HAZs for both 2001 and 2011. Fig 1 suggests an almost parallel shift of the entire distribution of HAZs—in effect, a distribution-neutral improvement in child growth outcomes. Fig 2 displays predicted HAZs by child’s age. Such plots inform two important characteristics of undernutrition in any given context: The extent to which undernutrition is the result of small size at birth (as captured by the intercept in Fig 2), which in turn would imply that maternal malnutrition is an important constraint; and the extent to which undernutrition is the result of postnatal growth faltering (the slope of Fig 2), which tends to be most acute from around 6 months of age to around 24 months of age (age 6 months coincides with the introduction of solid foods and liquids other than breast milk and with increased mobility and hence exposure to disease). There is a fairly uniform improvement in child growth outcomes at all ages, implying that most of the improvement in undernutrition in Nepal is the result of larger birth sizes and hence improved maternal nutrition (which is difficult to observe). Over time, however, there is also some small flattening out of the growth-faltering process from 6 months to 24 months of age, which may be attributable to improved diets, other care practices (such as vaccinations) or environmental factors (sanitation).

Descriptive statistics are provided in Table 2. Mean HAZs improved by approximately half a standard deviation from 2001 to 2011. Stunting fell from 56.6 percent to 40.0 percent during the same period, while severe stunting fell from 24.2 percent to just 14.6 percent.

**Table 2:** Trends in means for child nutrition and nutrition determinants, 2001, 2006, and 2011.

	Mean HAZ score	Stunting (%)	Severe stunting (%)	Asset index (1–10)	Maternal education (years)
2001	–2.17	56.6	25.3	2.1	1.5
2006	–1.90	49.6	18.7	3.8	2.6
2011	–1.66	40.1	14.60	5.1	3.7
*Change 2001–2006*	*0*.*51*	*–16*.*5*	*–10*.*7*	*3*.*0*	*2*.*3*
	Paternal education (years)	4 or more antenatal care visits (%)	Iron during pregnancy (%)	Born in hospital (0/1) (%)	All vaccinations (0/1) (%)
2001	4.3	8.5	22.1	9.2	52.4
2006	5.3	16.0	58.3	19.3	64.5
2011	5.8	28.6	78.5	36.3	69.8
*Change 2001–2006*	*1*.*5*	*20*.*1*	*56*.*4*	*27*.*0*	*17*.*4*
	Preceding birth interval (years)	Open defecation (% of village)	Water source—tubewell (%)	Water source—piped (%)	Women’s decisionmaking (%)
2001	3.9	75.1	37.7	35.4	12.1
2006	4.2	56.9	41.5	36.2	18.7
2011	4.6	42.3	31.4	48.3	23.5
*Change 2001–2006*	*0*.*7*	*–32*.*8*	*–6*.*3*	*12*.*9*	*11*.*4*

Source: Authors’ construction.

Notes: HAZ = height-for-age *z* score. Stunting (%) refers to HAZ<-2 and severe stunting to HAZ<-3.

Table 2 also shows trends in the explanatory variables in our models. The mean asset index score rose significantly (143%), corroborating the rapid growth in household income observed in household surveys (S1 Text). Women’s education improved rapidly from a low base. Health changes were even more striking (S2 Text). The proportion of children whose mothers received four or more ANC visits during pregnancy roughly tripled, and hospital births increased by almost 300 percent from a very low base, while the percentage of children older than 6 months who were fully vaccinated increased from slightly more than half to more than two-thirds. Demographic changes were also sizeable, with a significant decrease in fertility (measured in our models as birth order) and increase birth intervals, improvements which have previously been linked with nutrition outcomes [28, 29]. The prevalence of open defecation declined quickly, from 75.1 to 42.3 percent. There were more modest changes in water supply, with an increase in piped water of 13 percentage points. Finally, there was a marked change in women’s decisionmaking, though by the end of the sample the average woman had a say in only one of four possible decisions, suggesting gender inequality is still a significant problem.

Table 3 reports our basic regression results for the pooled sample. Column 1 reports an ordinary least squares model of HAZs against the set of explanatory variables listed in Table 3 as well as basic controls for age, location, and birth order effects (which are omitted for the sake of brevity). A one-unit change in the 0–10-scale asset index predicts a 0.04 standard deviation change in HAZs, suggesting that the growth gap between children from the poorest and richest households is about 0.40 standard deviation.

**Table 3:** Determinants of child growth in pooled regression models.

Regression number	1	2	3
Dependent variable	HAZs	Stunting	Severe stunting
Estimator	OLS	LPM	LPM
Asset index (1–10)	0.042**	–0.014**	–0.006**
	(0.007)	(0.003)	(0.002)
Maternal education (years)	0.028**	–0.007**	–0.003*
	(0.005)	(0.002)	(0.001)
Paternal education (years)	0.008*	–0.001	–0.003**
	(0.004)	(0.002)	(0.001)
4 or more antenatal care visits	0.092*	–0.036*	–0.008
	(0.036)	(0.014)	(0.010)
Iron during pregnancy	–0.029	0.004	–0.011
	(0.030)	(0.012)	(0.01)
Born in hospital (0/1)	0.200**	–0.062**	–0.017†
	(0.040)	(0.015)	(0.01)
All vaccinations (0/1)	0.110**	–0.031*	–0.045**
	(0.039)	(0.016)	(0.014)
Preceding birth interval (years)	0.031**	–0.010**	–0.006*
	(0.009)	(0.004)	(0.003)
Open defecation (%, village)	–0.151*	0.066**	0.036†
	(0.069)	(0.024)	(0.02)
Water—tubewell (0/1)	0.121**	–0.056**	–0.028*
	(0.045)	(0.017)	(0.012)
Water source—piped (0/1)	–0.032	0.002	0.003
	(0.035)	(0.014)	(0.010)
Women's empowerment (0–1)	–0.006	0.009	0.007
	(0.037)	(0.015)	(0.012)
Maternal height (centimeters)	0.055**	–0.018**	–0.012**
	(0.002)	(0.001)	(0.001)
*R*-squared	.316	.217	.139
*N*	9,341	9,341	9,858

Source: Authors’ estimates.

Note: OLS = ordinary least squares; LPM = linear probability model. Clustered robust standard errors are reported in parentheses. Notes: HAZ = height-for-age *z* score. Stunting (%) refers to HAZ<-2 and severe stunting to HAZ<-3. The regressions include a number of omitted controls, including period fixed effects, regional and agroecological fixed effects for 13 groups, an urban dummy, district-level population density, birth order dummies, dummy variables for religion and caste, a full set of month-specific child age dummy variables, dummy variables for various brackets of maternal age (in five year intervals), and Demographic Health Survey round dummy variables. See Table 2 for definitions of variables.

†Significant at the 10 percent level.

*Significant at the 5 percent level.

**Significant at the 1 percent level.

Maternal education has a commensurately large effect. The difference between a mother having no education and six years of education (completing primary) is about 0.17 standard deviations and about 0.34 standard deviations for completing secondary school. The impact of paternal education is about one-quarter the impact of maternal education and is significant at only the 10 percent level.

Receiving at least four ANC visits predicts a 0.09 standard deviation improvement in HAZs, but iron supplements for mothers during pregnancy are not associated with HAZs. Birth in a hospital is associated with almost 0.20 standard deviation improvement. Receiving all vaccinations also has a relatively large impact of 0.10 standard deviations.

Birth intervals have a significant but relatively modest effect, but birth order effects are insignificant. Open defecation at the village level also have a fairly modest marginal effect. Moving from a situation of 100 percent open defecation to 0 percent would improve HAZs by about 0.15 standard deviations. Water supplies yield the potentially surprising result that tube wells are associated with improved child growth outcomes, while piped water relative to more basic sources are not. The maternal decisionmaking index has no significant effect on child growth scores (controlling for women’s education, which has large marginal effects). Maternal height—an intergenerational effect rather than any policy-related impact—has a relatively large impact: every extra centimeter of height raises child HAZ by 0.05 standard deviations.

Qualitatively, many—but not all—of these basic results for HAZs carry over to the linear probability models (LPMs) estimated for stunting (regression 2) and severe stunting (regression 3), though the magnitudes of the effects vary between stunting and severe stunting. The asset index, maternal education, ANC visits, hospital births, birth intervals, and open defecation all have larger marginal effects on stunting than severe stunting. In contrast, paternal education significantly affects severe stunting but has no effect on stunting, and vaccinations seem to have a somewhat stronger association with severe stunting than stunting, suggesting that preventable diseases often cause severe forms of stunting. Consistent with the generally larger marginal effects in the stunting model, the explanatory power of the stunting model is substantially larger than the severe stunting model.

We assessed the robustness of our results in a number of ways, many of which are reported in the Supporting Information On-Line Supplementary Material (S3 Text). First, we used quantile regressions as an alternative means of exploring whether some factors are more important at the lower end of the distributions of HAZs or stunting scores. Quantile regressions allow us to explain variation around points of interest at the lower end of the HAZ distribution without having to resort to dichotomizing continuous variables. Results suggest that this approach—estimated at both the 25th and 50th quantiles—yields results similar to those in Table 3 (Table A in S3 Text).

Second, we estimated separate regression models for rural and urban areas and for Nepal’s three major agroecological zones: the plains (terai), hill and mountain regions (results available upon request). While we found small differences in coefficients very few of these were statistically significant. We also examined trends in explanatory variable for each agroecological zone. Encouragingly—given the inequalities that motivated the Maoist insurgency—the DHS suggest relatively similar social and economic improvements across the three regions.

Third, we estimated these models with the inclusion of district fixed effects (Table B in S3 Text). This made virtually no difference to any of the results, though it slightly altered the coefficients and standard errors attached to village-level open defecation (unsurprisingly, since only a handful of villages are measured for each district).

Fourth, we estimated models for younger children in the ranges 0–24 months and 0–12 months of age (Table C in S3 Text). One issue for older children is that mothers may struggle to remember utilization of health services several years ago, leading to attenuation bias in the estimate coefficients. Another issue is that DHS questions on toilet use and water sources ask about current use, not duration of use. If improved toilets were only adopted just prior to a survey round, then older children might not have been exposed to improved sanitation conditions in early life. This lack of prolonged exposure could also bias some coefficients down. Consistent with these expectations, results reported in our supplementary material show that the coefficient on open defecation increases substantially in absolute magnitude for younger age groups, suggesting our main results may be underestimating the contribution of sanitation to nutritional improvements in Nepal. The coefficients on our health indicators also change. The coefficients on antenatal care becomes insignificant, but the coefficients on “born in hospital” and “All vaccinations” become larger in magnitude. The implications of these changes are discussed further below.

Lastly, we used a bivariate correlation analysis to examine the stability of the relationships between dependent and independent variables over time. For those variables that were significant in the regression results reported in Table 3 we find little evidence of large changes in these relationships over time. We therefore conclude that there is little indication of substantial changes in these relationships over time.

**Fig 3:**
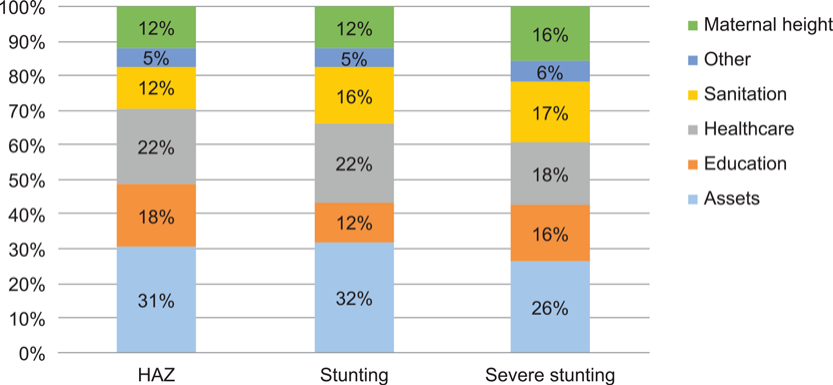
Contributions to predicted nutritional change by nutrition indicator. Source: Authors’ estimates. Note: HAZ = height-for-age *z* score.

We therefore use the results from Tables 2 and 3 to analyze the predicted sources of nutritional change over time, based on eq 2 in section 3. The results are reported in Table 4 and Fig 3. To see how these figures are derived, consider the second row of column 2 which reports the predicted change in HAZs, which is the mean change in assets from 2001 to 2011 multiplied by the coefficient of assets on HAZs from regression 1 of Table 3. This calculation suggests that improvements in assets from 2001 to 2011 predict a 0.13 standard deviation increase in child HAZs. In addition to the large predicted change resulting from asset accumulation, we observe sizeable contributions from maternal education (0.06), health indicators, reductions in open defecation, and maternal height. The bottom of Table 4 sheds light on the predictive power of these decompositions. For HAZ the model predicts a 0.40 standard deviation improvement, as opposed to an actual 0.51 HAZ improvement, which suggests the regression decomposition accounts for around 80 percent of the actual change observed from 2001 to 2011. The stunting decomposition performs similarly well, though the model for severe stunting has some less ability to explain change over time.

**Table 4:** Decomposing predicted changes in child growth outcomes, 2001 to 2011.

Dependent variable	HAZ score	Stunting (%)	Severe stunting (%)
Asset index (1–10)	0.13	–4.2	–1.8
Mother’s education (years)	0.06	–1.5	–0.7
Father’s education (years)	0.01		–0.5
4 or more antenatal care visits	0.02	–0.7	
Born in hospital	0.05	–1.7	–0.5
All vaccinations	0.02	–0.5	–0.8
Preceding birth interval	0.02	–0.7	–0.4
Open defecation	0.05	–2.2	–1.2
Water source—tubewell (%)	–0.01	0.4	0.2
Mother’s height	0.05	–1.6	–1.1
Predicted nutritional change	0.40	–13	–7
Actual nutritional change	0.51	–17	–11
Explanatory power of model (%)	79.3	77.7	62.2

Source: Authors’ estimates.

Note: HAZ = height-for-age *z* score. Stunting (%) refers to HAZ<-2 and severe stunting to HAZ<-3.

As noted above, restricting the HAZ model to younger children implied larger contributions from sanitation and health factors (Table C in S3 Text). Hence when we implement decompositions for children 0–24 months and 0–12 months we find that the overall contribution of health factors to nutritional change increases from 22.5% to almost 30%, and the contribution of sanitation from 12.5% to 18% (Table D in S3 Text). Moreover, these decompositions for younger children actually account for an increasing share of the observed change in HAZ over time. Indeed, the model for children 0–12 months explains 98.5 percent of the actual change in HAZ change over 2001–2011.

## Conclusions

This paper sought to understand rapid improvements in child nutrition in a remarkable success story. Despite large-scale conflict and political instability, Nepal achieved reduced stunting prevalence by 1.8 points per year from 2001 to 2011. Statistically, a number of factors convincingly account for this success.

First, much of the improvement in child nutrition stems from improvements in birth size, with only a modest improvement in postnatal nutrition trajectories. Second, asset accumulation emerges as an important factor across all nutritional indicators. Interestingly, asset accumulation was very rapid, which is consistent with rapid growth in household income (S1 Text). Third, consistent with many other studies, educational improvements appear to be important. Specifically, though, we find only maternal education predicts improved child nutrition outcomes in Nepal. Fourth, revolutionary improvements in access to healthcare have played a large role in Nepal, with potentially important lessons for other developing countries. Major government programs have explicitly targeted ambitious improvements in antenatal, neonatal, and postnatal care through rapid expansion of health extension workers as well as financial incentives. Fifth, Nepal has achieved sizeable gains in sanitation, specifically in the reduction of open defecation. Indeed, it is possible that our model underestimates the contribution of improved sanitation because the DHS questions on toilet use do not identify when specific practices were adopted. Multi-country experimental evidence suggests that the impacts of toilet adoption are about twice as large as those estimated in this paper [30].

The multi-dimensional and multi-sectoral nature of this change starkly emphasizes the point that rapid nutritional change seems chiefly to stem from simultaneous social and economic progress on multiple fronts [8–11; 31–32]. Indeed, our results for Nepal are strikingly similar to an analogous decomposition of nutritional change for Bangladesh [31], with the main except being a somewhat greater contribution of health services in Nepal. Together, these results suggest that emulating success stories requires overcoming some of the very challenging obstacles to achieving success on multiple fronts, including more effective inter-sectoral coordination.

## Supporting Information

S1 Text Construction of an Asset Index. (DOCX)

S2 Text Additional Descriptive Statistics. (DOCX)

S3 Text Additional Regression Results. (DOCX)

S4 Text STATA^TM^ Regression Code. (TXT)
