# Supplementary material for: Understanding the Rapid Reduction of Undernutrition in Nepal, 2001–2011
Source: PLoS One. 2015 Dec 23;10(12):e0145738. doi: 10.1371/journal.pone.0145738 (PMC4690594; doi:10.1371/journal.pone.0145738)
Supplement: S1 Text — (DOCX) [file pone.0145738.s001.docx]

# S1 Text. Construction of an Asset Index

One limitation of our analysis is that we were available to construct only a fairly simple asset index across the various rounds of the Demographic Health Survey using principal components analysis. Only six “asset” indicators were available for 2001, 2006, and 2011. These assets, and their respective factor loadings, were radio ownership (0.15), TV ownership (0.50), bicycle ownership (0.22), use of improved cooking fuel (kerosene, biogas, electricity; 0.46), basic flooring (–0.49), and household access to electricity (0.47).

Despite being a relatively parsimonious index, it appears that relatively little information is lost by using a 6-indicator index relative to the 9-component and 18-component indexes. The correlations between the 6-component index and the other two asset indexes are .9 and greater, and the 6-component index does as well in predicting height-for-age *z* scores as any of the other indexes. Moreover, although one might expect that the 6-component index is not as good at predicting differences at upper ends of the wealth distribution, we find no evidence of this (Figure A).

However, one inherent limitation of this index is that some of the components of the index may have effects on nutrition outcomes that are independent of wealth mechanisms. For example, cooking fuels may affect children’s susceptibility to acute respiratory infections, or radios and TVs may be important for receiving nutrition, health, and family planning messages. Another problem may be that some components of this index do not reflect household wealth so much as public investment in infrastructure, particularly electricity, which Figure B suggests was a major sources of total change in the asset index from 2001 to 2011.

Figure C shows HAZ-asset index relationships across the three rounds, and indicates that the relationships are quite stable. Table B looks at trends in an abbreviated version of the asset index (with just four assets), mean household income (from national surveys) and GDP per capita. The household surveys show rather rapid growth in mean income (almost 6% per annum), and the asset index (rescaled to 100) shows very rapid change from a very low base. However, GDP per capita shows much more moderate growth. So the rapid change in household income and assets is something of a disconnect from the national accounts data, perhaps because the latter hasn’t done a good job of accounting for growth in remittances.

## **Table A: Correlations between height-for-age *z* scores (HAZs) and three asset indexes with different numbers of components**

|  | HAZ | 6 components | 9 components | 18 components |
| --- | --- | --- | --- | --- |
| HAZ | — |  |  |  |
| 6 components | .24 | — |  |  |
| 9 components | .25 | .94 | — |  |
| 18 components | .26 | .90 | .93 | — |

Source: Authors’ calculations.

## **Figure A: The relationship between the 6-component asset index and the 18-component asset index**

Source: Authors’ LOWESS estimates from the 2011 rounds of the Demographic Health Survey (Nepal, MOHP, New ERA, and ICF International 2012).

## **Figure B: Sources of change in the 6-component asset index**

Source: Authors’ calculations.

## **Figure C. The relationship between the asset index and HAZ scores across rounds**

**

Source: Authors’ calculations.

## **Table B. Trends in alternative indicators of socioeconomic status**

|  | Asset index  (0-100) | Mean household income (2005 $) | GDP per capita  (2005 $) |
| --- | --- | --- | --- |
| 1996 | 14.98 | 37.53 | 261.83 |
| 2001 | 19.90 | n.a. | 298.74 |
| 2003 | n.a. | 53.96 | 301.80 |
| 2006 | 36.47 | n.a. | 325.80 |
| 2010 | n.a. | 68.06 | 375.92 |
| 2011 | 48.68 | n.a. | 384.45 |
| Annual percentage change | 14.99% | 5.81% | 3.12% |

Source: The asset index is rescaled from 0-100 to be comparable in scale to mean income. This is sourced from the various DHS rounds. Mean household income and GDP per capita are from the World Bank.
