# Supplementary material for: Understanding the Rapid Reduction of Undernutrition in Nepal, 2001–2011
Source: PLoS One. 2015 Dec 23;10(12):e0145738. doi: 10.1371/journal.pone.0145738 (PMC4690594; doi:10.1371/journal.pone.0145738)
Supplement: S2 Text — (DOCX) [file pone.0145738.s002.docx]

# S2 Text. Additional Descriptive Statistics

The tables below present some additional descriptive statistics on key variables (S3 Table), as well as more detailed statistics on utilization of health care services. S4 Figure shows iron supplementation by the number of ANC visits to justify our use of 4 or more ANC visits as our main indicator of antenatal care. S4 Table shows trends in a broader array of health utilization and health outcome indicators. Strikingly, there were impressive improvements across a wdie range of indicators. Table S5 explores the distributional indications of these improved health utilization outcomes by examining trends across asset index quartiles. The results suggest there were improvements across all asset quartiles. Finally, Figure s5 shows marked improvements in child feeding outcomes, as measured by the number of times children ate solid or semi-solid foods in the last 24 hours.

## **Table A. Summary statistics for key explanatory variables**

| Variable | Observations | Mean | Standard deviation | Minimum | Maximum |
| --- | --- | --- | --- | --- | --- |
|  |  |  |  |  |  |
| Asset index (1–10) | 16,994 | 2.25 | 2.76 | 0.01 | 10.00 |
| Maternal education (years) | 18,017 | 2.38 | 3.62 | 0.00 | 14.00 |
| Paternal education (years) | 17,840 | 5.08 | 4.11 | 0.00 | 14.00 |
| Number of antenatal care visits | 12,983 | 2.46 | 2.29 | 0.00 | 8.00 |
| Born in hospital (0/1) | 18,017 | 0.20 | 0.40 | 0.00 | 1.00 |
| All vaccinations (0/1) | 18,017 | 0.62 | 0.49 | 0.00 | 1.00 |
| Birth order | 18,017 | 2.92 | 2.02 | 1.00 | 15.00 |
| Preceding birth interval | 17,997 | 4.22 | 2.16 | 0.75 | 7.00 |
| Open defecation (% of village) | 18,017 | 0.60 | 0.35 | 0.00 | 1.00 |
| Water source—tubewell (0/1) | 17,002 | 0.36 | 0.48 | 0.00 | 1.00 |
| Water source—piped (0/1) | 17,002 | 0.39 | 0.49 | 0.00 | 1.00 |
| Women’s empowerment index (0–1) | 17,966 | 0.17 | 0.32 | 0.00 | 1.00 |
| Maternal height (centimeters) | 15,219 | 150.62 | 5.40 | 105.90 | 185.80 |

Source: Authors’ calculations.

Notes: See Table 1 in main text for definitions of variables.

## **Figure A. The probability of a mother’s receiving iron supplements by the number of antenatal care visits**

Source: Authors’ calculations.

## **Table B. Trends in health inputs and outcomes in Nepal, 1996 to 2011**

|  | 1996 | 2001 | 2006 | 2011 |
| --- | --- | --- | --- | --- |
| Antenatal care | | | | |
| Mothers with at least 1 visit (%) | 41.3 | 48.5 | 72.2 | 85.0 |
| Number of visits | 1.20 | 1.57 | 2.50 | 3.63 |
| 4 visits or more (%) | 6.3 | 9.0 | 16.0 | 30.6 |
| Iron supplements (%) | 10.8 | 15.6 | 41.3 | 61.5 |
| Tetanus shot (%) | 43.2 | 52.8 | 71.2 | 82.1 |
| Blood pressure taken (%) | Not available | 60.9 | 78.5 | 87.3 |
| Blood work done (%) | Not available | 27.5 | 27.4 | 44.8 |
| Urine tests done (%) | Not available | 29.2 | 31.4 | 54.8 |
| Neonatal Care | | | | |
| Born in health facility (%) | 7.8 | 9.4 | 17.2 | 36.1 |
| Assisted by a doctor (%) | 5.8 | 8.2 | 9.4 | 17.1 |
| Assisted by midwife/nurse (%) | 8.8 | 11.5 | 20.7 | 40.3 |
| Child health inputs and outcomes | | | | |
| Child received all vaccinations (%) | 30.1 | 53.4 | 64.4 | 70.0 |
| Child received vitamin A (%) | 25.7 | 74.0 | 86.3 | 79.5 |
| Diarrhea in past 2 weeks (%) | 57.6 | 40.4 | 24.3 | 27.0 |
| Fever in past 2 weeks (%) | 40.7 | 31.5 | 18.1 | 18.0 |
| Cough in past 2 weeks (%) | 48.1 | 40.4 | 18.3 | 20.8 |
| Under-5 mortality rate (per 1,000) | 139 | 108 | 79 | 62 |

Source: Authors’ calculations.

## **Table C. Trends in health service utilization by asset index quartiles, 2001 to 2011 (in percentages)**

|  |  | Asset quartiles | | | |
| --- | --- | --- | --- | --- | --- |
|  | Year | Quartile 1 | Quartile 2 | Quartile 3 | Quartile 4 |
| 4 antenatal care visits | 2001 | 2.9 | 5.0 | 17.9 | 28.5 |
|  | 2011 | 12.8 | 16.9 | 30.0 | 51.5 |
|  |  |  |  |  |  |
| Medical birth | 2001 | 3.0 | 5.0 | 15.0 | 35.0 |
|  | 2011 | 12.0 | 20.0 | 39.0 | 64.0 |
|  |  |  |  |  |  |
| All vaccines | 2001 | 46.0 | 59.0 | 57.0 | 62.0 |
|  | 2011 | 63.0 | 70.0 | 71.0 | 73.0 |

Source: Authors’ calculations.

## **Figure B. Trends in child feeding frequency, 2001 to 2011**

Source: Authors’ calculations.

Note: CI = confidence interval.

## **Figure C. Nonparametric estimates of the relationship between height-for-age *z* scores and continuous variables**

| Asset index | Maternal education | Paternal education |
| --- | --- | --- |
|  |  |  |
| Open defecation, village level | Child birth order | Preceding birth interval |
|  |  |  |

Source: These are local polynomial smoothing predictions with 95 percent confidence intervals estimated from the 2001 (Nepal, MOHP, New ERA, and ORC Macro 2002), 2006 (Nepal, MOHP, New ERA, and ICF International 2007), and 2011 (Nepal, MOHP, New ERA, and ICF International 2012) Demographic Health Surveys.
