# Supplementary material for: Understanding the Rapid Reduction of Undernutrition in Nepal, 2001–2011
Source: PLoS One. 2015 Dec 23;10(12):e0145738. doi: 10.1371/journal.pone.0145738 (PMC4690594; doi:10.1371/journal.pone.0145738)
Supplement: S3 Text — (DOCX) [file pone.0145738.s003.docx]

# S3 Text. Additional Regression Results

This supplement presents some additional regression results to provide robustness checks on the results present in the main text. Table A presents quantile regressions that minimize absolute deviations around different points in the HAZ distribution, namely the 50^th^ and 25^th^ percentiles, which correspond to -2.1 standard deviations and -2.95 standard deviations respectively. Table B presents regression results using district fixed effects. Table C presents compares regression results for children 0-59 months, 0-24 months and 0-12 months, and Table D reports corresponding decomposition results for these age brackets.

## **Table A. Quantile regression estimates of HAZ scores**

| Regression number | 1 | 2 |
| --- | --- | --- |
| Dependent variable | HAZ | HAZ |
| Estimator (percentile) | Quantile | Quantile |
| Quantile value | 50th = –2.10 | 25th = 2.95 |
|  |  |  |
| Asset index (1–10) | 0.042** | 0.032** |
|  | (0.008) | (0.009) |
| Maternal education (years) | 0.024** | 0.026** |
|  | (0.005) | (0.006) |
| Paternal education (years) | 0.007† | 0.013** |
|  | (0.004) | (0.005) |
| Number of antenatal care visits | 0.038** | 0.027** |
|  | (0.009) | (0.010) |
| Iron during pregnancy | –0.074* | –0.031 |
|  | (0.037) | (0.042) |
| Born in hospital (0/1) | 0.188** | 0.172** |
|  | (0.043) | (0.050) |
| All vaccinations (0/1) | 0.056 | 0.110** |
|  | (0.038) | (0.043) |
| Preceding birth interval | 0.041** | 0.022* |
|  | (0.010) | (0.012) |
| Open defecation (% of village) | –0.161** | –0.214** |
|  | (0.058) | (0.067) |
| Water source—tubewell (0/1) | 0.095* | 0.090* |
|  | (0.044) | (0.050) |
| Water source—piped (0/1) | –0.047 | 0.003 |
|  | (0.035) | (0.040) |
| Women’s empowerment (0–1) | –0.015 | –0.040 |
|  | (0.042) | (0.048) |
| Maternal height (centimeters) | 0.059** | 0.056** |
|  | (0.003) | (0.003) |
|  |  |  |
| *N* | 9,347 | 9,347 |

Source: Authors’ estimates.

Note: Standard errors are reported in parentheses. The regressions include a number of time-invariant controls, including period fixed effects, regional and agroecological fixed effects for 13 groups, an urban dummy, district-level population density, birth order dummies, dummy variables for religion and caste, a full set of month-specific child age dummy variables (except in the maternal BMI regression), dummy variables for various brackets of maternal age (in five year intervals), and Demographic Health Survey round dummy variables. See Table 1 in main text for definitions of variables. †Significant at the 10 percent level. *Significant at the 5 percent level. **Significant at the 1 percent level.

## **Table B. The determinants of child growth in a pooled regression model with district fixed effects**

| Regression number | 1 | 2 | 3 |
| --- | --- | --- | --- |
| Dependent variable | Height-for-age *z* score | Stunting | Severe stunting |
| Estimator | OLS | LPM | LPM |
|  |  |  |  |
| Asset index (1–10) | 0.046** | –0.015** | –0.008** |
|  | (0.007) | (0.003) | (0.002) |
| Maternal education (years) | 0.024** | –0.006** | –0.002† |
|  | (0.005) | (0.002) | (0.001) |
| Paternal education (years) | 0.009* | –0.002 | –0.004** |
|  | (0.004) | (0.002) | (0.001) |
| 4 or more antenatal care visits | 0.090* | –0.036* | –0.005 |
|  | (0.036) | (0.015) | (0.010) |
| Iron during pregnancy | –0.027 | 0.002 | –0.01 |
|  | (0.030) | (0.012) | (0.009) |
| Born in hospital (0/1) | 0.185** | –0.060** | –0.018† |
|  | (0.040) | (0.015) | (0.010) |
| All vaccinations (0/1) | 0.100** | –0.031* | –0.045** |
|  | (0.038) | (0.015) | (0.013) |
| Preceding birth interval (years) | 0.013* | –0.004† | –0.003* |
|  | (0.005) | (0.002) | (0.002) |
| Open defecation (%, village) | –0.130† | 0.071** | 0.029 |
|  | (0.075) | (0.025) | (0.022) |
| Water—tubewell (0/1) | 0.071 | –0.035† | –0.024† |
|  | (0.058) | (0.021) | (0.014) |
| Water source—piped (0/1) | –0.008 | 0.001 | –0.002 |
|  | (0.034) | (0.014) | (0.010) |
| Women’s empowerment (0–1) | 0.013 | 0.003 | 0.004 |
|  | (0.037) | (0.015) | (0.012) |
| Maternal height (centimeters) | 0.054** | –0.017** | –0.012** |
|  | (0.002) | (0.001) | (0.001) |
|  |  |  |  |
| *R*-squared | .316 | .217 | .139 |
| *N* | 9,341 | 9,341 | 9,858 |

Note: OLS = ordinary least squares; LPM = linear probability model. Clustered robust standard errors are reported in parentheses. The regressions include a number of omitted controls, including period fixed effects, regional and agroecological fixed effects for 13 groups, an urban dummy, district-level population density, birth order dummies, dummy variables for religion and caste, month-specific child age dummy variables (except in the maternal body mass index regression), dummy variables for various categories of maternal age, district fixed effects, and Demographic Health Survey round dummy variables. See Table 1 in main text for definitions of variables. †Significant at the 10 percent level. *Significant at the 5 percent level. **Significant at the 1 percent level.

## **Table C. The determinants of child growth for different age ranges of children**

| Regression number | 1 | 2 | 3 |
| --- | --- | --- | --- |
| Dependent variable | Height-for-age *z* score | Height-for-age *z* score | Height-for-age *z* score |
| Estimator | OLS | OLS | OLS |
| Age range | 0-59 months | 0-24 months | 0-12 months |
|  |  |  |  |
| Asset index (1–10) | 0.042*** | 0.030*** | 0.045*** |
|  | (0.007) | (0.011) | (0.016) |
| Maternal education (years) | 0.028*** | 0.033*** | 0.027*** |
|  | (0.005) | (0.007) | (0.010) |
| Paternal education (years) | 0.008** | 0.010 | 0.010 |
|  | (0.004) | (0.006) | (0.008) |
| 4 or more antenatal care visits | 0.092** | 0.068 | -0.059 |
|  | (0.036) | (0.054) | (0.085) |
| Iron during pregnancy | -0.029 | -0.019 | -0.073 |
|  | (0.030) | (0.041) | (0.062) |
| Born in hospital (0/1) | 0.200*** | 0.237*** | 0.297*** |
|  | (0.040) | (0.059) | (0.091) |
| All vaccinations (0/1) | 0.110*** | 0.149*** | 0.281*** |
|  | (0.039) | (0.053) | (0.092) |
| Preceding birth interval (years) | 0.031*** | 0.039*** | 0.018 |
|  | (0.009) | (0.014) | (0.020) |
| Open defecation (%, village) | -0.151** | -0.184* | -0.245* |
|  | (0.069) | (0.098) | (0.130) |
| Water—tubewell (0/1) | 0.121*** | 0.115* | 0.074 |
|  | (0.045) | (0.064) | (0.090) |
| Water source—piped (0/1) | -0.032 | -0.026 | 0.038 |
|  | (0.035) | (0.046) | (0.069) |
| Women’s empowerment (0–1) | -0.006 | 0.018 | 0.036 |
|  | (0.037) | (0.059) | (0.095) |
| Maternal height (centimeters) | 0.055*** | 0.058*** | 0.059*** |
|  | (0.002) | (0.004) | (0.005) |
|  |  |  |  |
| *R*-squared | 0.316 | 0.317 | 0.223 |
| *N* | 9341 | 4963 | 2557 |

Note: OLS = ordinary least squares; LPM = linear probability model. Clustered robust standard errors are reported in parentheses. The regressions include a number of omitted controls, including period fixed effects, regional and agroecological fixed effects for 13 groups, an urban dummy, district-level population density, birth order dummies, dummy variables for religion and caste, month-specific child age dummy variables (except in the maternal body mass index regression), dummy variables for various categories of maternal age, and Demographic Health Survey round dummy variables. See Table 1 in main text for definitions of variables. †Significant at the 10 percent level. *Significant at the 5 percent level. **Significant at the 1 percent level.

## **Table D. Decomposing predicted changes in child growth outcomes, 2001 to 2011, for different age ranges of children**

|  | 0-59 months | 0-24 months | 0-12 months |
| --- | --- | --- | --- |
|  |  | | |
|  | Predicted change (standard deviations) due to….. | | |
| Asset index (1–10) | 0.13 | 0.10 | 0.10 |
| Mother’s education (years) | 0.06 | 0.06 | 0.08 |
| Father’s education (years) | 0.01 | Not significant | Not significant |
| 4 or more antenatal care visits | 0.02 | Not significant | Not significant |
| Born in hospital | 0.05 | 0.10 | 0.10 |
| All vaccinations | 0.02 | 0.03 | 0.01 |
| Preceding birth interval | 0.02 | Not significant | Not significant |
| Open defecation | 0.05 | 0.07 | 0.07 |
| Water source—tubewell (%) | –0.01 | Not significant | Not significant |
| Mother’s height | 0.05 | 0.05 | 0.04 |
|  |  |  |  |
|  | Shares of predicted HAZ change (%) due to…. | | |
| Share due to assets | 32.5% | 23.4% | 24.5% |
| Share due to education | 17.5% | 15.6% | 18.6% |
| Share due to health factors | 22.5% | 31.2% | 28.5% |
| Share due to sanitation | 12.5% | 17.6% | 18.0% |
| Share due to other | 17.5% | 12.3% | 10.3% |
| Share due to all factors | 100.0% | 100.0% | 100.0% |
|  |  |  |  |
|  | Explanatory power of the model…. | | |
| Predicted nutritional change | 0.40 | 0.41 | 0.41 |
| Actual nutritional change | 0.51 | 0.49 | 0.41 |
| Ratio of predicted to actual (%) | 79.3% | 84.9% | 98.5% |

Source: Authors’ estimates.

Note: HAZ = height-for-age *z* score. See Table 1 in main text for definitions of variables.
